# The School Counselors Role in Supporting Teachers Working with Children who Have Experienced Trauma: Lessons Learned

**DOI:** 10.1007/s40653-024-00680-z

**Published:** 2025-01-07

**Authors:** Kristi L. Perryman, Timothy T.J., Hailey Thomas Frost

**Affiliations:** 1 University of Arkansas, 751 W. Maple, Fayetteville, AR 72701 USA; 2 Northern Illinois University, Dekalb, USA

**Keywords:** Trauma, Adverse childhood experiences, School counselor, Teachers

## Abstract

Teachers are dealing with the challenges of educating students who have been exposed to Adverse Childhood Experiences (ACEs) at an increasing rate (Brunzell et al., 2021; Mayor, 2021). Often their education has not prepared them for recognizing and mitigating the behaviors associated with ACE exposures. This article offers a review of current research regarding the role of the school counselor in supporting teachers. A case study provides insight into this support through a university partnership with a trauma-focused charter school. Suggestions are offered for teacher training and policy changes based on the author's experiences in working with a trauma-focused school. Specifically, there are many areas where school counselors can provide advocacy and support through efforts within their role in the areas of defining, managing, delivering, and assessing ASCA (2024). Partnering with a university can provide needed services for schools while simultaneously offering learning opportunities for students.

## Introduction

### Teaching Students who have Experienced Trauma

Teachers hold a unique role in the lives of their students. They serve as mentors and models while helping to build academic skills and support educational needs. They also facilitate peer interactions and offer emotional support (Alisic et al., 2012). Students who are exposed to Adverse Childhood Experiences (ACEs) might need more support from teachers. ACEs are potentially traumatic events that children ages 0–17 are exposed to that cover three areas: abuse, neglect, and household changes (Centers for Disease Control [CDC], 2020). ACEs differentiate from trauma due to ACEs being an exposure to a potentially traumatic event, whereas trauma is the response to the event (American Psychological Association, 2024). Students who are exposed to ACEs are more likely to have traumatic responses that have profound and far-reaching impacts on their ability to function effectively in school settings.

Felitti et al. (1998) revealed that children who experience high levels of adversity are more likely to encounter significant challenges in their academic and social-emotional development. These challenges can manifest in numerous ways, including lower academic performance and a higher likelihood of behavioral issues (Felitti et al., 1998). These students come to school bearing the burden of exposure to ACEs and traumatic responses that impact their ability to thrive in school. By providing teachers with trauma-informed training, they are better prepared to recognize and respond to students’ needs and to create learning environments that are safe and supportive (Willis & Nagel, 2015). School Counselors are well-positioned to offer this support.

Studies have shown that teachers are faced with the challenge of supporting children who have been exposed to ACEs but often feel ill-equipped to do so due to a lack of knowledge and training (Alisic et al., 2012; Brunzell et al., 2021; Mayor, 2021). Within a school setting, teachers may turn to the school counselor for support and guidance on how to best support these students. School counselors possess specialized training in mental health services and trauma response but are also perfectly positioned to consult and collaborate with teachers, guardians, administrators, and other specialists outside the school setting to provide needed services. This article will review current research regarding the status of trauma-informed training for teachers, the role of the school counselor in supporting teachers, and offer suggestions for training and policy changes based on the author's experiences in working partnership between a university and a trauma-focused school.

### Adverse Childhood Experiences

Thousands of children in the United States have experienced traumatic events. The Maternal Children's Health Bureau (MCHB) includes questions about adverse childhood experiences (ACEs) in their annual National Survey of Children's Health, completed by tens of thousands of families. The most recent available data shows that at least 15% of the child population in the United States has two or more exposure to ACEs (America’s Health Rankings, 2022). The more ACE exposures a child has, the more likely there will be adverse health outcomes for them during childhood and adulthood (CDC, 2020; Clarkson Freeman, 2014; Felitti et al., 1998; Ray et al., 2020).

Additionally, children who belong to a non-dominant group (race and sexual orientation) are more likely to be exposed to ACEs compared to those who belong to the dominant group (MCHB, 2022). This includes an increased risk of living in poverty and exposure to violence, abuse, neglect, and household dysfunction than children of the dominant racial group (Patterson et al., 2018; Post et al., 2019). This statistic is consistent across race and sexual orientation. Children who are part of some non-dominant racial identities (Black, Hispanic, and Multiracial) all have higher averages of ACEs compared to children who are White and Asian (Merrick et al., 2018). In addition to race, children who identify within the LGB population have a higher average of ACEs compared to children who identify as heterosexual (Merrick et al., 2018).

As mentioned, children who have been exposed to ACEs may experience trauma symptoms that interfere with their ability to learn (Alisic, 2012; Brunzell et al., 2018). The impact of ACEs on students is not limited to academic performance but extends to their behavior and social interactions. Children who have been exposed to ACEs have difficulty with coping skills and emotional regulation (Gruhn & Compas, 2020). They have a higher likelihood of experiencing externalized symptoms like those of Attention-Deficit/Hyperactivity-Disorder (ADHD) and other aggressive and disruptive behaviors (Clarkson Freeman, 2014; Fox et al., 2015; Fuller-Thomson & Lewis, 2015; Jimenez et al., 2017; Ray et al., 2020; Ray et al., 2022; Schoonover & Perryman, 2023). These behaviors can disrupt the learning environment, making it challenging for the affected students, their peers, and teachers.

The higher the number of ACEs elementary students have experienced, the higher the risk of poor school attendance, behavioral issues, and inability to meet academic standards (Blodgett & Lanigan, 2018). Students with high ACE scores are more likely to have lower grades, increased absenteeism, decreased school engagement, and higher dropout rates (Bethell et al., 2017; Crouch et al., 2019; Morrow & Villodas, 2017; Stempel et al., 2017). Teachers play a vital role in supporting these students who have experienced ACEs, helping to assist them in increasing attendance, decreasing behaviors in schools, meeting academic standards, and increasing school engagement. For teachers to support students in the classroom who have experienced ACEs more effectively, they need appropriate training and support.

### Teacher Preparation and Needs Worldwide

A review of the research emphasized the need for teachers to receive more education related to working with children who have experienced trauma to increase their knowledge regarding ways to provide emotional support to children, knowing when to refer and whom to refer to, as well as where the boundary is with their role as teacher versus counselor (Alisic, 2012; Alisic et al., 2012). Although previous research has highlighted the need to prepare teacher candidates to work with students who have experienced trauma, only five states have teacher licensure requirements that include the word trauma and training in trauma-informed pedagogy, and only nine states mandate training in identifying ACEs (Reddig & VanLone, 2022).

There is a lack of information on what trauma-informed training looks like for teachers (Brown et al., 2022a, 2022b; Reddig & VanLone, 2022; Thomas et al., 2019). Although there is limited literature on the training of teachers in trauma-informed practices, it could be included within their classroom management course or during their student-teaching experiences (Brown et al., 2022a, 2022b). Teachers in the United States reported feeling “somewhat prepared” to work with students who have experienced trauma and fear they do not have enough strategies or support to help these students (McClain, 2021). In addition to lacking the needed training to identify and mediate trauma behaviors in their classroom, teachers have also expressed struggling with their emotional reactions to students’ disclosure of trauma, especially if they have experienced trauma in their own lives. The emotional labor associated with vicarious trauma can lead to burnout and a decrease in teacher well-being (Alisic, 2012; Brunzell et al., 2018; Kim et al., 2021; Mayor, 2021). There is a need for more research regarding how teachers in the United States receive training in trauma-informed practices.

The need for trauma-informed training for teachers is universal. Teachers are salient in children's learning, social and cognitive development, and overall well-being (Willis & Nagel, 2015). Studies from the Netherlands (Alisic et al., 2012), Australia (Brown et al., 2022a, 2022b; Brunzell et al., 2021), Sudan and South Sudan (Heltne et al., 2020), New Zealand (Berger et al., 2016), and Canada (Mayor, 2021) have emphasized the important role that teachers serve in aiding children who have been exposed to ACEs. These studies have also highlighted teachers' experiences in working with children who have been exposed to ACEs and depict the need for additional training for teachers (Alisic et al., 2012; Anderson et al., 2022a, 2022b; Berger et al., 2016; Brown et al., 2022a, 2022b; Brunzell et al., 2021.) Recognizing the universal need for integrating trauma-informed practices for teachers is paramount and underscores school counselors' crucial role in contributing to students' holistic well-being.

With the prevalence and impact of trauma on student's academic success, educators have a responsibility to intervene. There has been a growing recognition of the importance of infusing teacher training programs with trauma-informed content to better equip teachers to meet the diverse needs of students affected by trauma. Little has been published on the role of the school counselor in supporting teachers regarding working with students who have experienced ACEs. Trauma-informed school initiatives have gained traction in response to the profound impact of trauma on students' academic and social-emotional development. These initiatives are designed to create safe, supportive environments that address the needs of trauma-affected students. Several initiatives have emerged aiming to integrate trauma-informed practices into teacher education curricula (Anderson et al., 2022a, 2022b; Berger et al., 2016; Hutchison, 2019; Plumb et al., 2016). One study examined the impact of a multi-pronged trauma-informed professional development for teachers (Anderson et al., 2022a, 2022b). This study showed that implementing a trauma-informed professional development program improved compassion satisfaction and decreased secondary traumatic stress for educators and highlighted the importance of the school counselor's role in the process (Anderson et al., 2022a, 2022b). Another study evaluated the effectiveness of a school-based resiliency program for teachers who experience secondary traumatic stress, demonstrating the ability of the program to decrease posttraumatic stress and secondary traumatic stress (Berger et al., 2016).

Studies have also indicated that trauma-informed school initiatives can positively impact academic achievement for students. For example, research by Dorado et al. (2016) found that schools that implemented trauma-informed practices saw improvements in school personnel’s understanding of trauma, an increase in students' grades and attendance, and a decrease in external trauma-related symptoms from students. Schools that have adopted trauma-informed approaches have also reported decreases in behavioral issues and disciplinary referrals and an increase in student engagement and participation (Morton & Berardi, 2018). This may likely be due to these initiatives increasing teachers’understanding of the impacts of trauma and providing strategies for creating supportive environments (Dorado et al., 2016). The school counselor plays a crucial role in these initiatives, and they hold significant promise for improving the academic, behavioral, and social-emotional outcomes of students affected by trauma. These potential benefits underscore the importance of continuing to support trauma-informed practices in educational settings. School counselors play a pivotal role in the implementation of trauma-informed school initiatives. By providing professional development, collaborating with teachers and other school administrators, and offering direct support to students, school counselors aid in creating a supportive educational environment that addresses the needs of students affected by ACEs.

### School Counselors' Role in Supporting Students and Teachers in the United States

School counselors play a vital role in assisting with trauma response within school settings. Equipped with specialized training to be the mental health professional in their school, they are uniquely positioned to provide support to students affected by trauma and teachers affected by secondary traumatic stress. Their roles encompass intervention and prevention strategies aimed at addressing the emotional, social, and academic needs of students and teachers. Previous literature on the school counselor’s role in supporting students who have experienced trauma focuses on the importance of using evidence-based practices (Alvarez et al., 2022; Zyromski et al., 2022). This includes educating teachers about the prevalence and impact of ACEs and equipping them with strategies to create supportive and trauma-sensitive classroom environments (Alvarez et al., 2022; Herrenkohl et al., 2019; Martinez et al., 2020; Opiola et al., 2020; Reinbergs and Fefer, 2018; Rumsey & Milsom, 2019; Zyromski et al., 2022). Programs have been developed to aid current teachers in working with children’s symptoms of trauma through psychoeducation (Brunzell et al., 2018; Kim et al., 2021) and skill-building (Gormez et al., 2017; Hutchison, 2019; Kim et al., 2021; Opiola et al., 2020). Although completing this training is a duty that is often implemented by school counselors to create a trauma-informed school (American School Counselor Association, 2024a), new school counselors often feel they were not prepared in their graduate program to complete this role along with the many others their job entails (Wells, 2022). In this case, they may seek to collaborate with outside professionals on this topic and offer professional development opportunities school-wide.

The American School Counseling Association (ASCA) defines the role of the school counselor. In addition to providing direct services to students, they are also tasked with indirect services to assist with student achievement. Indirect services include consultation, collaboration, and referrals (American School Counselor Association, 2019). These services may include those defined by the Multitiered Systems of Support (MTSS) focusing on the social emotional, career, and academic development of students (ASCA 2021). Tier 1 emphasizes interventions specific to schoolwide programming as well as classroom instruction. Tier 2 is focused on small groups, consultation, and collaboration, as well as individual counseling. Tier 3 also focuses on consultation, collaboration, and the referral process. This may include working with the principal and teachers to identify and resolve issues related to students, consulting with teachers to offer strategies for helping students in the classroom and ideas to guardians to form a plan for student success and referring to outside resources such as mental health professionals for more in-depth services for students (ASCA, 2021).

ASCA has specifically highlighted the crucial role school counselors play in supporting students who have been exposed to ACEs (American School Counselor Association, 2024a). School counselors are trained in providing direct counseling services to those who have trauma symptoms as well as in crisis response and are ideally positioned as the mental health professionals in their school to assist with ensuring teachers, principals, and staff are trauma-informed. In ASCA’s position statement on *The School Counselor and Trauma-Informed* Practice, they highlight that the school counselor’s role includes the following:

“Create connected communities and positive school climates that are trauma-sensitive to keep students healthy and in school and involved in positive social networks...

Educate staff on the effects of trauma and how to refer students to the school counselor.

Promote a trauma-sensitive framework for policies, procedures, and behaviors to the entire staff.” (American School Counselor Association, 2024a, n.p.)

The importance of the school counselor’s role in specifically providing services for students who have been exposed to ACEs and have trauma symptoms is also stressed by the Council for Accreditation of Counseling and Related Educational Programs (Council for Accreditation of Counseling and Related Educational Programs, 2023). The school counseling specialty section highlights this by including “school counselor roles and responsibilities in relation to the school emergency management plans” and “techniques of social-emotional and trauma-informed counseling in school settings” (p.24–25).

The school counselor can address the areas suggested by both ASCA and CACREP by offering professional development to teachers and staff themselves or collaborating with others in the field to provide this training. They can provide classroom observations and strategies for responding to students who have trauma-related behaviors that interfere with learning. They are also in the best position to refer teachers to mental health professionals to process their trauma that may directly impact the students they work with. Lastly, they can take their knowledge and skills, specifically related to trauma, and assist administrators in creating school policies and procedures that can help best support students who have experienced trauma and enlist administrators in advocacy efforts for policy changes in their state and country.

The following case example illustrates a partnership between a university counseling program and a charter school for children who experienced trauma, with professional development and resources offered that benefitted both the school counseling program and the counseling students, meeting both ASCA and CACREP requirements.

### Case Example

A charter school was established in 2019 in the southern United States for children who have experienced trauma. This school is the first and only one of its kind in a state with the highest documented incidence of children with two or more ACEs in 2018 (Northwest Arkansas Children’s Shelter, 2024). The school's goal is to create a trauma-centered educational environment to help students heal and increase social emotional growth. A faculty member from a local university was asked to serve as a university partner for the planning of the school. An internal grant was obtained to purchase assessment tools. The faculty member was instrumental in the hiring of both the school counselor and school-based clinicians.

Initially, there were four grades, K-3, with 10 students per class, one teacher, and two paraprofessionals. Since the first year, the school has expanded to include six grades, K-5th, and maintained two adults to 10 child ratios. The school counselor consulted with a Conscious Discipline expert and planned professional development. All teachers hired before the first year's start were trained in Conscious Discipline, a trauma-informed, evidence-based program designed to equip teachers with the skills to discipline in a way that is conducive to social-emotional learning and self-regulation skills (Bailey, 2014). The program is student-focused and emphasizes the relationship between students and adults and includes a neurodevelopmental model for addressing student needs.

### Mental Health Professionals in the School

The counseling focus implemented by the school counselor and two school-based clinicians from an outside agency was Centered Play Therapy CCPT because it is an evidence-based approach for working with children who exhibit behaviors associated with trauma such as anxiety, self-concept issues, social-emotional challenges, developmental delays, disruptive behaviors, and domestic partner violence (California Evidence-Based Clearing House for Child Welfare (2019). It is also closely aligned with Conscious Discipline, with a focus on increasing social-emotional skills and self-control with an emphasis on the relationship. CCPT also has a plethora of research completed over a 70-year span and has shown effectiveness in schools with various populations (Lin & Bratton, 2015; Post et al., 2019; Ray et al., 2015). CCPT has also been successfully utilized for teacher training to improve student/teacher relationships (Blalock et al., 2024; Sepulveda et al., 2011). The school counselor, the school-based clinicians, and the intern had all completed 150 continuing education hours in play therapy, which included specific coursework and practice focused on CCPT. This offered a common ground for implementing the skills and language that the teachers learned from their Conscious Discipline training with the CCPT focus of the counseling staff. An example of this included the counselor's reframing misbehavior as trauma-related behavior, creating empathy and understanding. A CCPT model for setting limits was taught. The ACT model (Landreth, 2023), Acknowledges the child’s desire, Communicates the limit, and Targets an acceptable alternative, increasing the likelihood of regulation and self-control while conveying a sense of acceptance of the child.

### Collaborating with Outside Professionals for Support

The school counselor maintained a partnership with a local university, where all the counselors had been trained to maximize support for teachers. Throughout the first year, the university incorporated assignments in courses for counseling students to learn experientially while offering a needed service to the charter school. Students in the Crisis Counseling course interviewed each guardian from the school using the ACEs Expanded Questionnaire. This version included 25 questions (Ray et al., 2020) to assess the type of trauma the student experienced for the school counselor to inform classroom structure and needed counseling services for the child and their family. The ACEs Expanded Questionnaire was utilized due to it being more inclusive of adversities (expanded drug and alcohol use questions, family support, food insecurity, expanded emotional abuse, and expanded physical abuse) that a child could experience and provides some Likert scale responses.

The school counselor and principal contacted the guardians since they had an existing relationship and asked them to complete the ACEs to inform them of the needs of the students in their school. Teachers were not informed of the ACEs or results and only the school counselor and the counseling faculty had access to the scores to protect confidentiality. The school counselor illustrated the results in a graph for the administration to offer insight into the types of trauma students had experienced but no identifying data was shared. The school counselor and administrators met with each guardian of the student and discussed their trauma as well as trauma-related behaviors that were present at home and school. In addition to this, the university used the Social Emotional Assets and Resilience Scales (SEARS) with guardians, teachers, and students. Furthermore, the university administered the Trauma Symptom Checklist for Children with both parents and teachers. These added to the qualitative information gained from the interview to better understand potential trauma-related behaviors that students were exhibiting. The results of these assessments were shared with the teachers to gain a better understanding of their classroom and to view the behaviors as symptoms of trauma rather than misbehavior.

Through this partnership, professors and Ph.D. students from the university worked with the school counselor to create and conduct training for teachers and staff with an overview and language of CCPT and the neurological impact of trauma, creating a framework for understanding the impact of ACEs on classroom behaviors and learning. The half-day training was in person at the school and took place during the first week when the teachers returned for professional development, prior to students starting. This training reinforced what teachers had learned through the Conscious Discipline curriculum and offered insight into the therapeutic work the counselors would be doing with students. It also aimed to create empathy, encourage co-regulation, and offer consistency in acknowledging feelings, esteem building, relationship building, setting limits, etc. This training encouraged a common language and way of thinking about children who have experienced trauma to create a consistent and safe learning environment. The counseling staff at the school offered ongoing modeling when conducting classroom lessons or interventions in the classroom and throughout the school and offered support as needed to the teachers.

The school counselor also requested assistance in the development of a comprehensive school counseling program. School counseling students worked with the school counselor to create and implement a comprehensive school counseling program based on the ASCA National Model (2019). This included classroom and small group lessons focused on social-emotional resilience, self-regulation, and relational skills, to enforce the goals of conscious discipline and CCPT for children who have experienced trauma to reinforce the skills learned through Conscious Discipline and CCPT, supporting both students and teachers. University students served as consultants with the school counselor and administrator and co-authors of the comprehensive program.

A Ph.D. counseling student intern trained in both school counseling and play therapy was placed at the school for an internship. They assisted the school counselor in constructing lessons for the classrooms, managing crises, and conducting individual play therapy sessions with students, which resulted in their dissertation. This symbiotic partnership created opportunities for counseling students to learn and offer needed services that benefited the school and community. It also offered a common framework for understanding trauma-related behaviors and consistently addressing them throughout the school. In addition to having a partnership with a university to support the school, the school counselor and university assisted in policy changes to improve the school.

### Policy and Practice

Policy changes at the school were still being developed as the school is still new and experienced closure during the pandemic. Thus far changes have included requiring the ACEs as a part of the application for the charter school with only the principal and school counselor having access. This helped create classrooms in which not all children had experienced the same types of traumas and offering insight into the types of interventions (individual counseling, small group, classroom lessons).

Another policy change that was implemented by the school counselor to help support teachers was the addition of free counseling services for teachers with masters-level counseling interns from the university to address stress and burnout. A support group was also developed by the school counselor and facilitated by the school-based clinicians and university faculty to support faculty and staff. Because the school had an increasingly high burnout rate for teachers and paraprofessionals, a policy change was recommended to the school to create an interview protocol that specifically assessed applicants’ knowledge and capacity to deal with trauma-related behaviors, with opportunities to shadow current teachers.

The school counselor plays a significant role in training, supporting, and creating policies to support students who have been exposed to trauma. School counselors are one of the most qualified people in the school to train faculty and support students. It is a requirement of Council for Accreditation of Counseling and Related Educational Programs, (2023) that school counselors who attended an accredited school learn about trauma, its impacts, and their role within the school of supporting students and staff. Additionally, ASCA (2024) highlights the vital role that school counselors play in supporting students, educating staff, and promoting trauma-sensitive policies, procedures, and behaviors. This makes the role of the school counselor imperative in supporting students, educating teachers and staff, and collaborative efforts to create trauma-sensitive policies.

The school counselor in this study was in her third year of her profession, though the first in an elementary school. The school-based clinicians were new graduates. The efforts and implemented through this partnership better prepared them for their role not only at the school, but in the profession. Students completing practica and internships at the school also benefitted from the training, modeling, and hands on application that the partnership provided.

## Implications for School Counselors

### Training and Supporting Teachers

The school counselor, as the mental health professional in the school, can train teachers and staff as a part of their consulting role (American School Counselor Association, 2019). This section will offer examples from the school described in the case study as well as suggestions. School counselors can offer training regarding ACEs and evidence-informed interventions that can be used to support students who have been exposed to them (Anderson et al., 2022a, 2022b). This training can be done before the beginning of the school year as it was in the case study, or during professional development days throughout the year. It would be essential to include the neurological impacts of ACEs during this training so that teachers and staff can have a better understanding of what happens to students when they are exposed to ACEs as well as how to mitigate the effects.

The training for the school in the case study was conducted with teachers, paraprofessionals, and administrators before the first school year began. At the end of the first year, teachers reported that this was helpful but that they needed ongoing training and support aligning with research by Kinkead-Clark (2019). Although services were offered in terms of free tele-mental health counseling with master's level counseling interns from the university and follow-up training, the school encountered many challenges during the first year that interfered such as the high turnover rate with teachers and paraprofessionals. In the future, it could be helpful to include additional readings that teachers and staff can complete outside of the training. See Table 1 (in appendix B) for a list of books that can be provided to teachers and staff. They could even be added to the school library for teachers to have access.

The training in the case study included aspects related to self-care and a clear discussion and development of a personal wellness plan with the teachers. Brunzell et al., (2021) emphasize the need to support teachers in reducing their stress while also supporting them to improve their overall health and well-being. This can be completed by providing information on the importance of identifying coping skills to reduce stress, developing a healthy support system, receiving psychoeducation on ACES, developing mindfulness practices, and getting connected with their mental health resources (Anderson et al., 2022a, 2022b; Berger et al., 2016; Brunzell et al., 2021).

School administration can meet the need to support teachers in reducing stress as mentioned previously (Brunzell et al., 2021) by offering professional development, allowing mental health days from work, and offering support groups at the school as well as self-care opportunities such as yoga. Training should also be included specific to secondary trauma and burnout and stress to educate teachers and offer warning signs. Regular support groups for the teachers would be an excellent way to offer support, offering opportunities to process daily experiences and connect with others while also identifying with any countertransference they may be experiencing (Dorado et al., 2016; Eyal et al., 2019; Frydman & Mayor, 2017). The study discussed previously by Kinkead-Clark (2019) emphasized the need for ongoing support for the teachers rather than a one-time training. Monthly or weekly support group meetings by the school counselor or a mental health professional from outside the school could meet this need and increase the long-term benefits of the training. Partnering with a university for master's or Ph.D. level counseling students to facilitate CACREP requirements could be another way to continue the partnership that benefits the school and student counselors, protecting the school counselors' time with students and benefitting teachers as they may be more comfortable sharing with someone outside the school system.

As part of the training and the Assess component of American School Counselor Association, (2019), it is crucial for the school counselor to collect data from the teachers and staff. School counseling programs are data-driven and provide a rationale for their roles within the school. American School Counselor Association, (2019) describes and provides templates for the implementation of the Assess area of the national model in which school counseling programs work to analyze data to explore if the programs delivered assisted the success of students.

A needs assessment with students, teachers, and staff to assess what needs and current knowledge exist related to trauma would be helpful. Aragon et al. (2024) conducted a needs assessment for educators regarding implementing trauma-informed practices in school. Pre-and post-data collected from the completed training can inform the counselor about learning and changes that may be needed on a personal and systemic level (Aragon et al., 2024). This can be conducted after each lesson or periodically throughout the year to assess if the knowledge is retained. Lastly, it is vital for the school counselor to clarify in the training what their role in assisting with teacher and student trauma-related needs so that teachers and staff know to seek consultation from them as needed. Data collection and analysis can inform the school regarding needs and establish policies and procedures with administrators to support students exposed to trauma and the teachers who work with them.

In the case study above, informal feedback was obtained from teachers to gain information but not until the end of the first year. This should have been done during both the first and second semesters so that adjustments could have been made and support put into place and could be in the form of focus groups to glean more salient information, while assessments were administered at three points during the first two years, they were based on teacher observation of trauma behaviors and the social-emotional resiliency of students. More research would have been beneficial to better understand the impact on teachers and their needs. Using the data to help make improvements was challenging because of the high teacher turnover during those first two years. Frequently, a different teacher completed an assessment on a student on the second round than on the first, for example, which threatened the validity of the results.

### School Counselor's Role in Policy Changes

There are multiple ways that the school counselor can help implement policy changes and practice to ensure that teachers are trauma-informed and meet the growing need for student interventions. Examples from the school in this manuscript will be shared as well as suggestions for ways to impact policy change through assessment, trauma-sensitive policies, counseling curriculum, and lower teacher-to-student and school counselor-to-student ratios.

### Assessment

The importance of data collection was mentioned in the teacher training section. This data can directly inform policy changes. The school counselor can analyze the data and share it with the administrator, along with suggestions for policy changes. These may include the requirement of wellness plans for the teachers, with the school counselor coordinating services offered at the school such as mindfulness training and yoga.

In the case study, assessments were not included for faculty training but were for all students to assess for ACEs. Such a universal screening would be an important policy in schools focused on working with students who have experienced trauma. This could include the ACEs or Pediatric ACEs and Related Life Events Screener (PEARLS; Thakur et al., 2020). There are multiple considerations when including a trauma screener as a universal assessment. One, the guardian completing the assessment could be the person who has caused the trauma, so there could be a chance that all answers are not truthful. Second, it raises the issue of all school staff being mandated reporters, and they might need to report some of the responses. Some of these considerations have been written about, and it has been suggested by Amirazizi and colleagues (2022) that a school counselor works with key collaborators, uses a vital assessment, explains to the guardians why it is being used, and works with administrators and teachers to decide if data should be identified or de-identified. They also suggest screening for adversity and social-emotional health.

### Trauma Sensitive Policies

The school counselor should collaborate with administrators to create trauma-sensitive policies that focus on supporting students exposed to trauma and their teachers (American School Counseling Association, 2024b; 2019). The training provided by the school counselor can be the start of this cultural shift and buy-in from everyone in the school on these trauma-sensitive practices. In the case study, the school counselor and administrators ensured that all staff being hired understood the focus on supporting students who have experienced ACEs and creating a common language to do so. This information was shared in the job posting, continued in the job interview, and through the training that led up to the start of the school year. This assisted in creating a culture where all teachers, staff, and administrators understood the goals of the school. This step was essential to create this culture; however, there were some employees who had a tough time with the transition. That is where school policies reinforced the culture of the school, resulting in some teachers leaving and new ones being hired to align with policy.

The school in the case study had policies allowing students to have accommodations that do not necessarily include an Individualized Education Plan or 504 plan. Some examples included allowing the student the ability to leave the classroom and check in with the school counselor as needed, creating a calm-down corner for them, and allowing these students multiple opportunities to complete a task.

Another trauma-sensitive policy that benefitted students at the school in the case study stated they were not allowed to be sent home from school or excluded from recess regardless of their behavior. The school counselor can collaboratively create such a policy with administrators and teachers to shift from the exclusionary discipline in the school as a part of their indirect services through the Deliver component of American School Counselor Association, (2019). This would require the school counselor to utilize their advocacy skills and work with the whole school to create alternatives to exclusionary discipline for students, such as an alternative to the suspension program, Positive Behavior Interventions and Supports, and Restorative Justice, Collaborative and Proactive Solutions (Greene & Haynes, 2021). This shift could be difficult if a school is rooted in exclusionary discipline, and the school counselor would need to use data and research to support the use of alternative modes of discipline.

### Counseling Curriculum

Another way the school counselor can impact school policy change also falls under the Deliver section of the ASCA National Model (2019), as they provide direct services to students through classroom instruction. They can implement a social-emotional learning (SEL) curriculum and consult with teachers as part of indirect services to help them incorporate lessons one to three times a week (Perryman et al., 2020). SEL programs include interventions that can be implemented through both classroom lessons and small groups in schools. Zins and Elias (2007) defined SEL as “the capacity to recognize and manage emotions, solve problems effectively, and establish positive relationships with others” (p. 234). SEL programs promote effective learning and cognitive skills and skills to support successful interactions with peers and encourage prosocial actions over aggressive behavior through self-regulation and relational skills (Jones et al., 2017; Collaborative for Academic, Social, and Emotional Learning, 2019). Lerner (2022) suggested including activities in classroom lessons for students who have experienced trauma that focus on their problem behaviors, feelings, connection with others, creating solutions, and most importantly, making them feel safe. For an example of a classroom lesson for Kindergartners, see Appendix A.

Infusing SEL into the daily or weekly classroom curriculum can provide students who have been exposed to trauma the opportunity to develop their social-emotional skills faster than if the school counselor were to come in once a week to complete a classroom lesson (Perryman et al., 2020). The school counselor and Ph.D. intern at the school in the case study created thirty-minute classroom SEL lessons focused on mindfulness, conflict resolution, coping skills, gratitude, feeling identification, empathy, and careers. They quickly realized that adaptations were needed in terms of both the lessons and the amount of time to conduct them. They then decided to have both the school counselor and intern complete lessons together, shorten lessons and make them more interactive. This direct service to students is crucial to developing relationships and to SEL. Students at this school needed more SEL instruction than the school counselor alone could provide. The school counselor could offer curriculum suggestions for teachers to adopt that best fit their teaching style, and the student needs in their classroom to increase these needed skills daily.

### Lower Ratios

Finally, the school counselor can impact policy change by advocating with administrators for lower teacher-to-student ratios, which are key quality indicators in programs for early childhood education (Perlman et al., 2017). While there is an absence of research on staff-to-student ratios in trauma-focused schools or in working with students who have experienced ACEs, the researchers' experience indicated that even smaller ratios were needed for effectively working with this population. Smaller ratios could be beneficial in preventing teacher burnout and providing needed support to multiple dysregulated students in a classroom. Studies have indicated that smaller ratios improve child outcomes in areas such as socioemotional and cognitive functioning (Bowne et al., 2017; Glass & Smith, 1979; Duncan, 2003). One way this could be accomplished is by including more paraprofessionals in the classroom. While this would increase the financial expenditure of the school, the potential gains may be well worth it in terms of teacher burnout and well-being. Recruiting teachers with adequate trauma training is difficult as it is constantly having to replace those who leave. In the author's experience, these issues increase both the financial and emotional strain on the school. An added benefit could be the students' increased well-being, as the 3–10 adult-to-student ratio at the trauma-focused school in the case study seemed small, but it was not enough for children who have experienced multiple ACEs.

In addition to advocating for smaller ratios for teachers-to-students, the school counselor can advocate for smaller school counselor-to-student ratios. The most recent data indicates that the national average school counselor-to-student ratio is 385 to 1 (American School Counselor Association, 2024b), which is much higher than the school in the case study. The school counselor can advocate for smaller caseloads in a few manners. First, they can advocate for hiring additional school counselors to support students. Especially if the school counselor is working at a school with high needs and a high percentage of students with ACE exposures. Second, they could advocate for one of the school counselors in the school to have a smaller caseload of high-needs students who have exposure to ACEs. This could provide students with additional support without hiring a new school counselor. Although both options could have negative impacts on the budget or caseload for other school counselors, the focus on students with exposure to ACEs and who have high needs could decrease the stress from students, teachers, and staff and have a positive impact on the school climate.

## Conclusion

This article reviewed current research regarding the inclusion of trauma-informed training for teachers, the role of the school counselor in supporting teachers, and offered suggestions for training and policy changes based on the author's experiences in working with a trauma-focused school and existing literature. Many lessons were learned from the university's partnership with the trauma-focused school in the case study presented. There were many challenges described as the very nature of a school with all students having experienced trauma creates the potential for teacher, administrator, and school counselor burnout. The behaviors exhibited by the students and trying to modify them to create an environment for learning takes a huge emotional toll.

There were also many benefits to the school as faculty and staff had a common toolbox for identifying trauma-related behaviors, redirecting students, and setting limits as well as a language to use in working with guardians. They were able to consult with university professors trained in trauma as needed and had many services that they otherwise would not have. The information gained from the ACE was helpful as they informed classroom lessons, screening for small groups, etc. Having counselors who all had the same training created a strong working team. The benefits to university students were tremendous in terms of firsthand experience with students, administrators, teachers, guardians, etc. They gained confidence in their skills and familiarity with the school environment and culture.

There are many areas where school counselors can provide advocacy and support through efforts within their role in the areas of defining, managing, delivering, and assessing (American School Counselor Association, 2024a). Finally, partnering with university counseling programs can be mutually beneficial for student counselors and the school. Counseling students from CACREP accredited programs are trained in crisis and trauma and can offer extra support to the school counselor while gaining hands-on experience. Administrators should consider partnering with local university counseling, social work, and education programs to utilize interns trained in trauma in the classrooms to maximize the benefits to their students. This could provide needed experience to the interns and in turn offer a needed service to students in the classroom (Anderson et al., 2022a).

## Data Availability

NA.

## Appendix B

See Table 1

**Table 1:** Trauma Related Books for Teachers

Author	Book Title
Bruce D. Perry & Maria Szalavits	The Boy Who Was Raised as a Dog: And Other Stories from a Child Psychiatrists Notebook
Bessel van der Kolk	The Body Keeps the Score: Brain, Mind, and Body in the Healing of Trauma
Nadine Burke Harris	The Deepest Well: Healing the long-Term Effects of Childhood Trauma and Adversity
Kristin Souers & Pete Hall	Fostering Resilient Learners: Strategies for Creating a Trauma-Sensitive Classroom
Patricia A. Jennings	The Trauma-Sensitive Classroom: Building Resilience with Compassionate Teaching
